# One-year outcome of brolucizumab for neovascular age-related macular degeneration in Japanese patients

**DOI:** 10.1038/s41598-024-52747-4

**Published:** 2024-01-30

**Authors:** Satoru Inoda, Hidenori Takahashi, Ryota Takahashi, Yuto Hashimoto, Hana Yoshida, Rika Tsukii, Hironori Takahashi, Hidetoshi Kawashima, Yasuo Yanagi

**Affiliations:** 1 Department of Ophthalmology, Jichi Medical University, 3311-1 Yakushiji, Shimotsuke-shi, Tochigi, 329-0431 Japan; 2 Department of Ophthalmology and Micro-Technology, Yokohama City University, Yokohama, Japan

**Keywords:** Corneal diseases, Macular degeneration, Retinal diseases

## Abstract

A new anti-vascular endothelial growth factor agent, brolucizumab, was approved by the United States Food and Drug Administration in 2019. We evaluated whether brolucizumab reduces the treatment burden of neovascular age-related macular degeneration (nAMD) after switching by examining 1-year treatment outcomes in a real-world setting. This retrospective single-institution study included 107 consecutive eyes with nAMD treated with brolucizumab. Among these eyes, 30 with treatment-naïve nAMD and 77 treated with other anti-VEGF agents for more than a year were included. All eyes were managed using a treat and extend (TAE) or modified TAE regimen. The last injection intervals at 52 weeks were 12.9 and 12.1 weeks in the treatment-naïve and switch therapy groups, respectively. Among switch therapy group patients whose pre-switch injection intervals were shorter than 120 days (n = 62 eyes), the injection interval was significantly longer after the switch than before, with a mean difference of 2.7 weeks (*P* < 0.0001). Intraocular inflammation events occurred in 2 and 7 treatment-naïve and switch therapy patients, respectively. In conclusion, brolucizumab might reduce the treatment burden in patients who required the injection of other anti-VEGF agents with a 120-day interval or shorter, despite a relatively high discontinuation rate due to intraocular inflammation.

## Introduction

Macular neovascularisation (MNV) of the eye that develops with neovascular age-related macular degeneration (nAMD) causes exudative/haemorrhagic changes at the macular area, which leads to severe irreversible vision loss. Anti-vascular endothelial growth factor (anti-VEGF) injection, the first-line therapy for nAMD, is the only treatment option to prevent vision loss; however, there are still some challenges in real-world practice. The most significant burden to patients and physicians is the need for frequent injections, along with their associated high cost.

A new anti-VEGF agent, brolucizumab, was first approved by the United States Food and Drug Administration in 2019 and then by the relevant bodies in various countries^1–4^. The HAWK and HARRIER trials indicated that brolucizumab appears to have a more prolonged effect and may reduce the treatment burden compared with aflibercept^5^. Regarding its real-world effectiveness, some short-term treatment reports, mostly from Japan, have thus far reported relatively favourable results^6–10^.

These studies encouraged the use of brolucizumab. However, there still is a need to balance out the potential risk of intraocular inflammation (IOI)^11^ and the effectiveness of brolucizumab for its appropriate use. Recently, Liegl RG, et al. reported that patients who switched to brolucizumab had a median treatment interval extension of about 3 weeks at 12 months^12^. However, in Asian countries, where polypoidal choroidal vasculopathy (PCV) is highly prevalent in patients with MNV^13,14^, this result does not always apply to Asians. Thus, long-term treatment outcomes with a relatively large number of patients are required. To fill this gap, we herein report the 52-week treatment outcomes of 107 eyes with nAMD treated with brolucizumab in a real-world setting, with a particular focus on the treatment interval in the treat and extend (TAE) regimen in both treatment-naïve and switch therapy patients.

## Results

### Patients’ demographic characteristics

Table 1 shows the demographic characteristics of the patients included in this study. Ninety-eight patients and 107 eyes were followed via a TAE or modified TAE regimen. Thirty eyes in 27 patients and 77 eyes in 71 patients were in treatment-naïve and switch therapy groups, respectively. The central subfield foveal thickness (CST) of the treatment-naïve group was significantly thicker than that of the switch therapy group (*P* = 0.0.013). Other patients’ characteristics were not significantly different between the two groups. For the MNV classification, type 1 MNV and pachychoroid polypoidal choroidal vasculopathy (PCV) accounted for 24 and 46 eyes, respectively, in the switch therapy group, whereas type 1 MNV accounted for 16 eyes of the treatment-naïve group.

**Table 1:** Patient characteristics.

	All	Treatment-naïve	Switch therapy	*P*-value
Patients (eyes)	98 (107)	27 (30)	71 (77)	
Age, years (SD)*	75.2 (8.8)	72.5 (7.8)	76.2 (9.0)	0.064
Male, n (%)^†^	69 (66.7)	18 (66.7)	51 (70.4)	0.62
BCVA, LogMAR (SD)*	0.30 (0.29)	0.24 (0.27)	0.32 (0.30)	0.20
CST, µm (SD)*	324 (180)	412 (279)	290 (106)	0.0013
MNV types^†,‡^	40:9:1:55:2	16:3:0:10:1	24:6:1:46:1	0.24
Anti-VEGF agent before brolucizumab		Aflibercept: 72 Ranibizumab: 5		

*BCVA* best-corrected visual acuity, *CST* central subfield foveal thickness, *MNV* macular neovascularisation, *PCV* polypoidal choroidal vasculopathy.

*One-way ANOVA; ^†^Pearson’s chi-square test; ^‡^MNV types were classified into type 1 MNV, type 2 MNV, mixed types 1 and 2, PCV and type 3 MNV.

In the switch therapy group, the anti-VEGF agents before brolucizumab switching were aflibercept in 72 eyes and ranibizumab in 5 eyes.

### 52-week outcomes of best-corrected visual acuity, CST and IOI

As summarised in Table 2, both best-corrected visual acuity (BCVA) and CST significantly improved in the treatment-naïve group (*P* = 0.0037 and 0.0007, respectively) and neither BCVA nor CST significantly changed (*P* = 0.46 and 0.17, respectively) in the switch therapy group. IOI events occurred in 2 and 7 eyes, and the times to IOI occurrence after the first brolucizumab injection were 25.5 ± 29.4 weeks and 10.3 ± 9.5 weeks in the treatment-naïve and switch therapy groups, respectively (Table 2). In those nine patients, six patients showed iridocyclitis, three patients showed opacity of the vitreous, and four patients showed vasculitis without occlusion. Note that there is some overlap in these findings. These nine eyes (8.4%) were treated with injection of triamcinolone acetonide into sub-Tenon’s capsule, and there were no cases of residual visual dysfunction in the observation period. Patients who experienced IOI did not continue brolucizumab injections and received aflibercept injections after IOI.

**Table 2:** Change in BCVA and CST.

	Treatment-naïve		*P*-value	Switch therapy		*P*-value
	Baseline	After 52 weeks		Baseline	After 52 weeks	
BCVA, LogMAR (SD)	0.241 (0.274)	0.122 (0.229)	0.0037	0.321 (0.300)	0.342 (0.364)	0.46
CST, µm (SD)	412.2 (279)	236 (112)	0.0007	290 (106)	282 (111)	0.17

Paired *t*-tests.

*BCVA* best-corrected visual acuity, *CST* central subfield foveal thickness.

The BCVA recovered to the pre-IOI level after 52 weeks (*P* = 0.81).

### Injection intervals

**Figure 1:**
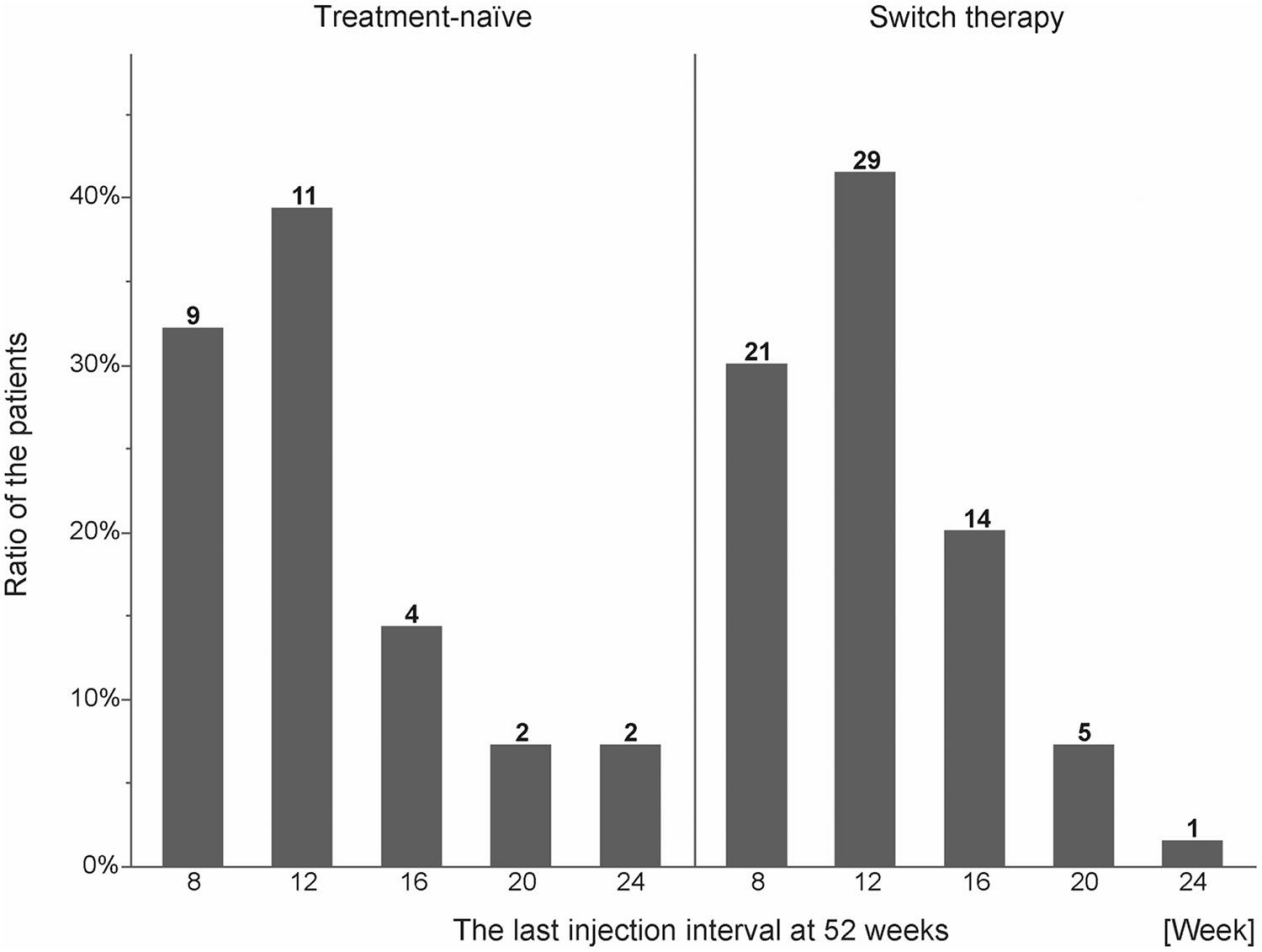
Last injection intervals at 52 weeks. Eleven eyes (39.3%) in the treatment-naïve group and 29 eyes (41.4%) in the switch therapy group had a 12-week or shorter injection interval at 52 weeks.

The mean last injection intervals were 12.9 ± 5.1 and 11.9 ± 3.7 weeks and the numbers of injections were 6.2 ± 1.5 and 5.2 ± 1.2 in the treatment-naïve and switch therapy groups, respectively. Figure 1 shows the last injection interval at 52 weeks. In 11 eyes (39.3%) in the treatment-naïve group and 29 eyes (41.4%) in the switch therapy group, the injection interval was 12 weeks or shorter at 52 weeks. In the treatment-naïve group, two patients had intervals of 24 weeks or longer.

There were 77 eyes in the switch therapy group. To compare the change in the injection interval, the 77 eyes were divided into two groups based on a pre-switch injection interval shorter than 120 days or longer than 120 days. After the further exclusion of eyes with IOI, the former group included 62 eyes and the latter included eight eyes. The pre-switch injection intervals were 4 to 8 weeks, 8 to 12 weeks, and 12 to 16 weeks in 20 eyes, 29 eyes, and 13 eyes, respectively.

The mean pre-switch injection interval of the other eight eyes was 34.6 ± 16.0 weeks.

**Figure 2:**
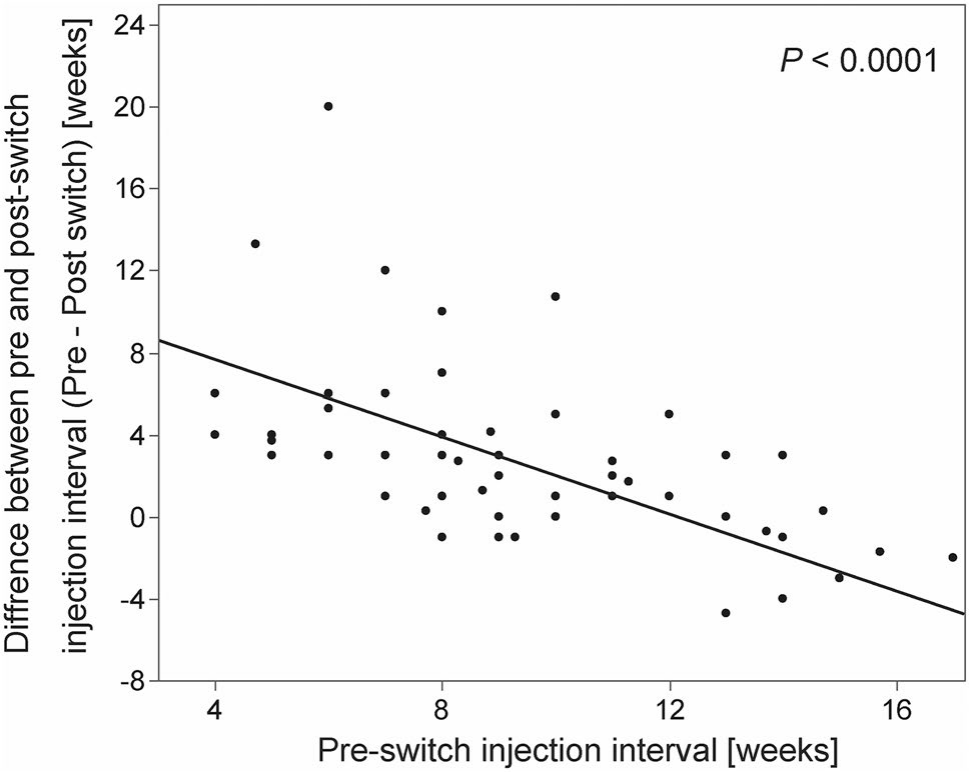
Association between the pre-switch injection interval and the difference between the pre- and post-injection intervals. A shorter pre-switch injection interval was associated with a significantly prolonged post-injection interval.

In the entire switch therapy group, the mean pre-switch injection interval was 12.1 ± 10.0 weeks and the mean last post-switch injection interval was 12.1 ± 3.7 weeks (*P* = 0.97). Among eyes with pre-switch injection intervals shorter than 120 days, the mean pre-switch injection interval was 9.2 ± 3.1 weeks and the mean post-switch injection interval was 11.9 ± 3.8 weeks (*P* < 0.0001) (Table 3). The change between the pre-switch injection interval and post-switch injection interval was 0.037 ± 10.1 weeks overall but 2.7 ± 4.3 weeks in the eyes of individuals with pre-switch injection intervals shorter than 120 days. Multivariable analysis of the difference between the post- and pre-switch injection intervals showed that a shorter pre-switch injection interval was associated with a significantly longer post-switch interval (*P* = 0.0002). A scatter plot of the difference between the pre- and post-switch injection interval against the pre-switch injection interval is shown in Fig. 2. In the treatment-naïve group, the last injection interval at 52 weeks was 12.9 ± 5.1 weeks.

**Table 3:** Mean injection intervals changes in the switch therapy group.

	Injection interval change (SD), weeks	Pre-switch injection interval (SD), weeks	Post-switch injection interval (SD), weeks	*P*-value
All (70 eyes)	0.037 (10.1)	12.1 (10.0)	12.1 (3.7)	0.97
Within 120 days (62 eyes)*	2.7 (4.3)	9.2 (3.1)	11.9 (3.8)	< 0.0001
Greater than 120 days (8 eyes)*	− 20.8 (16.8)	34.6 (16.0)	13.8 (3.2)	0.010

*Paired *t*-test.

*BCVA* best-corrected visual acuity, *CST* central subfield foveal thickness, *IOI* intraocular inflammation, *IVBr* intravitreal brolucizumab.

### Sample size calculation

A sample size calculation was not performed prior to the study. For the primary efficacy variable (treatment naïve group; change in BCVA from baseline to 52 weeks after switch, switching group; change in injection interval 52 week after switch), it was calculated that, in treatment naïve group, based on the results of a phase III clinical trial, a sample size of 25 is sufficient to detect a 6.9-letter improvement in visual acuity at 52 weeks with a standard error of 0.61 letters at a power of 0.8 and a significance level of 0.05. In switch group, sample size of 21 patients is sufficient to detect a difference in injection intervals of 3 weeks after switching with a standard deviation of 4 weeks at a power of 0.90 and a significance level of 0.05.

## Discussion

The current study showed that BCVA and CST significantly improved in a treatment-naïve group but did not significantly change in a switch therapy group. Patients in the switch therapy group with a pre-switch injection interval shorter than 120 days (n = 62 eyes) had a significantly longer injection interval after the switch than before, with a mean difference of 2.7 weeks. This result is consistent with a previous report in German and also with a previous short term report from Japan^12,15^.

In the current study, the post-switch injection interval was 3 weeks longer than the pre-switch injection interval in patients whose pre-switch injection interval was shorter than 120 days. Moreover, a shorter pre-switch injection interval led to a significantly prolonged post-switch injection interval. Although the post-switch injection interval was not longer than the pre-switch injection interval in patients whose pre-switch injection interval was longer than 120 days, this might be due to the chronicity of nAMD. In eyes with nAMD showing an exudative change from MNV with an interval of 120 days or more, the MNV activity may be low but chronic and the irregular exudative changes might not be related to the half-life of anti-VEGF agents. On the other hand, in eyes with nAMD that show an exudative change from MNV with an interval shorter than 120 days, MNV activity may be relatively high and these patients may need a more effective treatment, such as VEGF suppression. Brolucizumab may particularly reduce the treatment burden of patients with nAMD who needed frequent treatment with other anti-VEGF agents. This persistence and effectiveness of brolucizumab might be because of its high affinity for VEGF and its low molecular weight^16^.

In the treatment-naïve group, the mean last injection interval at 52 weeks was 12.9 ± 5.1 weeks and the mean number of injections was 6.2. In the ALTAIR study, which was a randomised, open-label, phase 4 study of the efficacy and safety of intravitreal aflibercept with TAE regimens (2- and 4-week adjustments) in Japanese patients with nAMD and which allowed a minimum interval of 8 weeks and a maximum interval of 16 weeks^17^, the mean last injection intervals at 52 weeks were 10.7 weeks and 11.8 weeks for the 2- and 4-week groups, respectively, and the mean numbers of injections were 7.2 and 6.9, respectively. In the current study, brolucizumab treatment for nAMD might have a similar effect to reduce the treatment burden compared with aflibercept treatment for nAMD.

Previous reports of the short-term outcomes of intravitreal brolucizumab injection for PCV showed regression rates of the polypoidal lesion of 78.9% and 78.6%^7,8^. We could not evaluate polypoidal lesion regression using indocyanine green angiography (ICGA) and we tried to evaluate the polypoidal lesion regression rate using OCT and colour fundus photography after 52 weeks of treatment (see Supplementary Table S2 and the Supplementary Note online). The regression rate was about 80%, which was similar to the rates that were reported previously using brolucizumab for 1 year^9^. In addition, in the switch therapy group, regression was obtained for over 80% of polypoidal features that remained with anti-VEGF agent treatment other than brolucizumab. Although these polypoidal regression rates were evaluated by OCT and fundus photographs but not ICGA, these rates using intraocular brolucizumab monotherapy for PCV are higher than those of intraocular aflibercept monotherapy or ranibizumab monotherapy with or without photodynamic therapy (PDT) combination therapy, which are about 55.4% (aflibercept monotherapy), 69.3% (ranibizumab with PDT) and 34.7% (ranibizumab monotherapy)^18–20^. Regression of polypoidal lesions would be a better outcome and reduce the treatment burden of PCV^10,21^, and brolucizumab might be a better treatment option for PCV compared with ranibizumab, aflibercept and PDT.

The IOI occurrence rate and time to IOI onset were not significantly different between the treatment-naïve and switch therapy groups. Nine eyes (8.4%) developed IOI during the 52-week treatment period. Some recent reports have provided data on IOI after intravitreal injection of brolucizumab; this condition requires prompt discontinuation and anti-inflammatory treatment and the reported IOI rates ranged from 2.4 to 22%^6,7,11,22,23^. Although the occurrence rate is in line with that of previous reports, the present work reveals no additional risk factors. IOI after brolucizumab intravitreal injection would cause severe visual loss due to retinal vasculitis, and further study of a larger number of patients is thus urgently needed.

This study has some limitations that merit discussion. These limitations are common to this type of prospective analysis involving a single centre: all patients were Japanese, and the study might be influenced by regional differences among the Japanese population. However, because it is a single-centre study, there was suitable control over the re-treatment criteria. Second, the number of eyes classified as each MNV subtype was small, and the efficacy for each MNV subtype was not adequately evaluated. In addition, our treatment regimen of modified TAE allowed a little subretinal fluid, and we are unable to discuss the retinal dry rate after the 52-week treatment. Finally, we did not evaluate the polypoidal regression rate using ICGA. Although the combination of OCT scans and colour fundus photography helps us to evaluate polyp features, these findings did not completely reflect polyp presence. It is possible that the polypoidal regression rate reported in this study is overestimated, and further studies are needed to confirm these findings.

In conclusion, we evaluated the efficacy of brolucizumab in Japanese nAMD patients. Although we do not know the risk factors or detailed causes of IOI and need to use brolucizumab with caution, the current study revealed that brolucizumab might reduce the treatment burden in people with treatment-naïve nAMD and in people who needed anti-VEGF agent intravitreal injection intervals shorter than 120 days.

## Methods

### Ethical approval and consent

This study was approved by the institutional review board of Jichi Medical University (JICHI20-127) and adhered to the tenets of the 1964 Declaration of Helsinki and its later amendments or comparable ethical standards. The study procedures followed the guidelines of the institution, and all patients provided informed consent prior to the procedures.

### Procedure

There were 214 eyes of 195 nAMD patients that treated with brolucizumab 6 mg with an at least 52-week follow-up at Jichi Medical University Hospital between May 2020 and February 2022. Patients who were treated with brolucizumab as the first treatment comprised the treatment-naïve group, whereas those who had a history of anti-VEGF therapy before brolucizumab injection were the switch therapy group. Consecutive patients who undergoing treatment with brolucizumab were provided comprehensive drug information regarding brolucizumab, and patients who voluntarily opted to switch to brolucizumab were included in this observational study. In switch therapy group, to compare the injection intervals before and after the brolucizumab injections, patients who were followed up via the as-needed regimen were excluded from the current study. This retrospective study included 107 eyes of 98 patients.

As a complete baseline clinical examination, all patients underwent an ophthalmic assessment with refraction, BCVA testing, slit lamp biomicroscopy with or without contact lenses, indirect ophthalmoscopy, colour fundus photography, fluorescein angiography (FA), ICGA and swept-source OCT. The diagnostic criteria for MNV were based on fundus examination, fundus photography, FA/ICGA or swept-source OCT (performed by HT, SI and RT). Colour fundus photography was obtained using a commercially available fundus camera system (VX-10; Kowa Co., Ltd., Nagoya, Japan). FA/ICGA was performed using a confocal scanning laser ophthalmoscope (Heidelberg Retina Angiography; Heidelberg Engineering, Heidelberg, Germany). Cross-sectional images of the macula were obtained using swept-source OCT (DRI OCT Triton; Topcon, Tokyo, Japan).Multimodal imaging was used to classify MNV based on the previously reported criteria^24^, with MNV classified as type 1, pachychoroid PCV, non-pachychoroid PCV, type 2, mixed type 1 and 2, and type 3 MNV.

### Inclusion and exclusion criteria

This study included nAMD patients that treated with brolucizumab 6 mg with an at least 52-week follow-up at Jichi Medical University Hospital between May 2020 and February 2022. Novartis had no role in the design or funding of this study. Inclusion criteria were Japanese ethnicity, aged ≥ 50 years, active MNV lesions secondary nAMD affecting the central subfield (the circular area within 1 mm diameter around the foveal center on imaging), onset of vison loss within six months prior to the initiation of treatment. The diagnosis of nAMD was based on the diagnostic criteria for nAMD in Japan. The diagnostic criteria for MNV were based on fundus examination, fundus photography, fluorescein angiography/ICGA, spectral domain-OCT, or swept source-OCT (DRI OCT-1 Triton; Topcon Corp, Tokyo, Japan). With swept- source-OCT, 9-mm radial scans through the foveal center were performed. In this classification, patients are diagnosed with nAMD if they have (1) choroidal neovascularization, (2) serous pigment epithelial detachment, (3) hemorrhagic pigment epithelial detachment and/or (4) fibrotic scar. Diagnostic were performed by retinal specialists. (S.I., H.T., and R.T.)

In switch therapy group, after through information was given to regarding the risk and benefits of intravitreal brolucizumab injections, we sought informed consent from patients who chose to receive brolucizumab. To compare the injection intervals before and after the brolucizumab injections, patients who were followed up via the as-needed regimen were excluded from the current study. In addition, patients with less than 52 weeks of nAMD treatment history were excluded.

### Injection intervals

We assessed the injection intervals to compare the change in the interval between before and after switchover in the switch therapy group. The injection interval just before switchover was defined as the pre-switch injection interval. The post-switch injection interval, occurring after 52 weeks, was defined as period between injections after 52 weeks from the first brolucizumab injection. This definition was used to mitigate the impact of the previous treatments on subsequent injections.

### Treatment design

Treatment-naïve patients were followed up via a TAE regimen (interval, 4 weeks). Switch therapy group patients were followed up via the same regimen, modified TAE or as needed. Switch therapy group patients were treated with ranibizumab (Lucentis; Genentech, San Francisco, CA) or aflibercept (Eylea; Regeneron, Tarrytown, NY).

The TAE regimen comprised the following steps.

1. An induction phase, during which patients received three monthly brolucizumab injections
2. A TAE phase, comprising the fourth and subsequent injections, during which patients received brolucizumab injection at every visit and the period until the next injection was extended by 4 weeks at a time, up to a maximum interval of 16 weeks, depending on disease activity^25^. If any signs of recurrence appeared, the treatment interval was shortened by 4 weeks at a time, to a minimal interval of 8 weeks, until a dry macular was achieved. This was defined as complete resolution of intraretinal and subretinal fluid without new haemorrhage and no change in pigmented epithelial detachment. If no sign of recurrence was observed after the second 16-week injection, the proactive treatment was interrupted, and the patients were seen again after 2 months to check the exudative change.

The difference between the TAE and modified TAE regimens in the current study was that, in the modified TAE regimen, maintenance and cessation were added, not just an extension or shortening of the injection interval. In the TAE and modified TAE regimens, disease activity was defined as a new retinal haemorrhage, presence of fluid on OCT or a change in serous pigmented retinal detachment. In the modified TAE regimen, fluid was defined as the presence of any intraretinal fluid and any subretinal fluid of more than 100 µm in height at the subfoveal centre. Subfoveal subretinal fluid of 100 µm or less or any subretinal fluid elsewhere was tolerated when it appeared by itself and, under these conditions, the treatment period was maintained^26–28^. Treatment should focus on vision gains rather than PED resolution because there is no apparent correlation between anatomical and functional improvement in most eyes with PED and nAMD^29,30^. We thus considered changes in serous PED to be tolerable and upheld the treatment interval with the change of serous PED alone.

Based on previous reports that visual acuity can be maintained even when the treatment interval is extended to 16 weeks^17^, we set the maximum treatment interval to 16 weeks. In addition, considering studies indicating that recurrence is less likely to occur when dry macula was confirmed at an interval of 16 weeks (rather than 12 weeks), we devised a treatment-free interval protocol^31–39^.

In this study, the injection interval at switching was instructed to maintain their regular visit intervals (± 1 week) after the initial IVBr injection, regardless of the presence or absence of exudative changes on that day.

### Statistical analysis

Statistical analysis was performed using JMP Pro software version 16.2.0 (SAS Institute, Cary, NC). Categorical data were assessed using Pearson’s chi-square test or Fisher’s direct probability test, and continuous variables were assessed using Student’s *t*-test, paired *t*-test and one-way analysis of variance (ANOVA). Statistical significance was defined as *P* < 0.05. Because repeat tests were not performed, no correction for multiple comparisons was made.

### Supplementary Information

Supplementary Information.

## Data Availability

The datasets used and/or analysed during the current study are available from the corresponding author on reasonable request.
