# Supplementary material for: One-year outcome of brolucizumab for neovascular age-related macular degeneration in Japanese patients
Source: Sci Rep. 2024 Jan 30;14:2451. doi: 10.1038/s41598-024-52747-4 (PMC10827718; doi:10.1038/s41598-024-52747-4)
Supplement: Supplementary file 1 — Supplementary Information. [file 41598_2024_52747_MOESM1_ESM.docx]

**Supplementary Information for**

**One-year outcome of brolucizumab for neovascular age-related macular degeneration in Japanese patients**

Satoru Inoda, MD, PhD; Hidenori Takahashi, MD, PhD; Ryota Takahashi, MD; Yuto Hashimoto, MD; Hana Yoshida, MD; Rika Tsukii, MD; Hironori Takahashi, MD; Hidetoshi Kawashima, MD, PhD; Yasuo Yanagi, MD, PhD

**Contents**

Supplementary Table S1. IOI occurrence and time to IOI onset.

Supplementary Table S2. Change in polypoidal lesion features on OCT' and fundus photographs.

Supplementary Note

Supplementary Table S1. IOI occurrence and time to IOI onset.

|  | All | Treatment-naïve | Switch therapy | P-value |
| --- | --- | --- | --- | --- |
| IOI, (%)* | 9 (8.4) | 2 (6.7) | 7 (9.1) | 0.68 |
| Time to IOI onset after IVBr, week (SD) ^‡^ | 13.6 (14.9) | 25.5 (29.4) | 10.3 (9.5) | 0.22 |

^*^Pearson’s chi-square test; ^‡^one-way ANOVA.

IOI, intraocular inflammation; IVBr, intravitreal brolucizumab.

Supplementary Table S2. Change in polypoidal lesion features on OCT' and fundus photographs.

|  | Baseline | | | | |
| --- | --- | --- | --- | --- | --- |
|  | Positive for three features | Positive for two features | Sharp-peaked PED | Sub-RPE ring-like lesion | Orange nodule |
| Treatment-naïve | 9 (100%) | 9 (100%) | 9 (100%) | 9 (100%) | 9 (100%) |
| Switch therapy | 22 (44%) | 26 (52%) | 27 (54%) | 27 (54%) | 23 (46%) |
|  | After 52 weeks | | | | |
|  | Positive for three features | Positive for two features | Sharp-peaked PED | Sub-RPE ring-like lesion | Orange nodule |
| Treatment-naïve | 2 | 2 | 3 | 2 | 3 |
| Regression rate | 77.8% | 77.8% | 66.7% | 77.8% | 66.7% |
| Switch therapy | 4 | 9 | 10 | 12 | 5 |
| Regression rate | 81.8% | 57.7% | 63.0% | 55.6% | 78.3% |

PED, pigmented epithelial detachment; RPE, retinal pigment epithelium.

Patients who developed intraocular inflammation were excluded.

**Supplementary Note**

**Polypoidal lesion regression rate**

Fifty-five eyes (51.4%) had PCV in this retrospective study. Not all patients underwent ICGA before and 52 weeks after the first brolucizumab injection. Some PCV-specific features can be seen on colour fundus photographs or OCT, such as orange nodule or double-layer sign. A previous report showed high specificity for the detection of polyps with three of the following features: an orange nodule on colour fundus photography and sharp-peaked PED and a sub-RPE ring-like lesion on OCT [1]. We used these features to evaluate the polyp regression rate after the 52-week treatment period. Polyp regression rates were confirmed using the detection of both two features and three features.

The three features were seen before brolucizumab treatment in 9 and 22 eyes in the treatment-naïve and switch therapy groups, respectively. After 52 weeks of treatment with brolucizumab, these features remained in 2 and 4 eyes, giving regression rates of 77.8% and 81.8% in the treatment-naïve and switch therapy groups, respectively (Supplemental Table 3). With the two-feature criterion, the regression rates were 77.8% and 57.7% in the treatment-naïve and switch therapy groups, respectively.

**Reference**

1. Chong TKY, Sadda SR, Gemmy CCM, et al. Non-ICGA treatment criteria for Suboptimal Anti-VEGF Response for Polypoidal Choroidal Vasculopathy: APOIS PCV Workgroup Report 2 Ophthalmology Retina. 2021;5(10):945-953
